# Sero-prevalence of human immunodeficiency virus–hepatitis B virus (HIV–HBV) co-infection among pregnant women attending antenatal care (ANC) in sub-Saharan Africa (SSA) and the associated risk factors: a systematic review and meta-analysis

**DOI:** 10.1186/s12985-020-01443-6

**Published:** 2020-11-07

**Authors:** Hussein Mukasa Kafeero, Dorothy Ndagire, Ponsiano Ocama, Abdul Walusansa, Hakim Sendagire

**Affiliations:** 1 Department of Medical Microbiology, College of Health Sciences, Makerere University, P.O Box 7062, Kampala, Uganda; 2 Department of Medical Microbiology, Habib Medical School, Faculty of Health Sciences, Islamic University in Uganda, P.O Box 7689, Kampala, Uganda; 3 Department of Plant Sciences, Microbiology and Biotechnology, College of Natural Sciences, Makerere University, P.O Box 7062, Kampala, Uganda; 4 Department of Medicine, College of Health Sciences, Makerere University, P.O Box 7062, Kampala, Uganda

**Keywords:** Antenatal care, HBV–HIV co-infection, Pregnant mothers, Sub-Saharan Africa

## Abstract

**Background:**

There is plenitude of information on HIV infection among pregnant mothers attending antenatal care (ANC) in sub-Saharan Africa. However, the epidemiology of HBV–HIV co-infections in the same cohort is not clear despite the common route of transmission of both viruses. The aim of our study was to synthesize data on the prevalence of HBV–HIV co-infection among pregnant women attending ANC in Sub-Saharan Africa to assist in the design of public health interventions to mitigate the challenge.

**Methods:**

The study was done in tandem with the Preferred Reporting Items for Systematic Reviews and Meta-analyses (PRISMA) standards and the Cochran’s Q test, I^2^ statistics for heterogeneity and the prevalence were calculated using commercially available software called MedCalcs (https://www.medcalc.org). A random effect model was used to pool the prevalence since all the heterogeneities were high (≥ 78%) and P_het_ < 0.05 indicated significant heterogeneities. The risk factors and risk differences for HBV–HIV co-infection were analyzed. Any likely sources of heterogeneity were analyzed through sensitivity analysis, meta-regression and sub-group analysis. All analyses were done at 95% level of significance and a *P* < 0.05 was considered significant.

**Results:**

The overall pooled prevalence of HBV–HIV co-infection among pregnant mothers in sub-Saharan Africa was low 3.302% (95%CI = 2.285 to 4.4498%) with heterogeneities (I^2^) of 97.59% (*P* > 0.0001). Within regional sub group meta-analyses, West Africa had significantly higher prevalence of 5.155% (95% = 2.671 to 8.392%) with heterogeneity (I^2^) of 92.25% (*P* < 0.0001) than any other region (*P* < 0.001). Articles published from 2004–2010 had significantly higher prevalence of 6.356% (95% = 3.611 to 9.811%) with heterogeneity (I^2^) 91.15% (*P* < 0.0001) compared to those published from 2011 to 2019 (*P* < 0.001). The HIV positive cohort had significantly higher prevalence of HBV–HIV co-infection of 8.312% (95% CI = 5.806 to 11.22%) with heterogeneity (I^2^)94.90% (*P* < 0.0001) than the mothers sampled from the general population with a prevalence of 2.152% (95% CI = 1.358 to 3.125%) (*P* < 0.001). The overall and sub group analyses had high heterogeneities (I^2^ > 89%, *P* < 0.0001) but was reduced for South Africa (I^2^) = 78.4% (*P* = 0.0314). Age, marital status and employment were independent factors significantly associated with risk of HBV–HIV co-infection (*P* < 0.001) but not extent of gravidity and education level (*P* > 0.05). After meta-regression for year of publication and sample size for HBsAg positivity, the results were not significantly associated with HBV pooled prevalence for sample size (*P* = 0.146) and year of publication (*P* = 0.560). Following sensitivity analysis, the HBsAg pooled prevalence slightly increased to 3.429% (95% CI = 2.459 to 4.554%) with heterogeneity I^2^ = 96.59% (95% CI = 95.93 to 97.14%), P < 0.0001

**Conclusion:**

There is an urgent need for routine HBV screening among HIV positive pregnant mothers attending antenatal care in sub-Saharan Africa to establish the extent of HBV–HIV co-infection in this cohort. Future studies need to investigate the putative risk factors for HBV–HIV co-infection and prioritize plausible control strategies.

## Introduction

Sub-Saharan Africa (SSA) is the epicenter of many infectious diseases including Human Immunodeficiency Virus (HIV) and Hepatitis B Virus (HBV) [1, 2]. The world Health Organization (WHO) estimates that 70–95% of the adult population in sub-Saharan Africa is exposed to HBV with Hepatitis B Surface Antigen (HBsAg) sero-prevalence rates of 6–20% [3]. Similarly, more than 25.6 million people in SSA are living with HIV [4]. The HIV and HBV share the same route of transmission making co-infection with both viruses plausible. The global burden of HBV–HIV co-infection is estimated at 2.6 million people with prevalencies ranging from 10 to 25% in moderate endemic to high endemic regions of SSA [5]. The Viral interactions in HIV–HBV co-infection complicate antiviral therapy [6] and reduce the Cluster of Differentiation (CD) −4 count [7]. Patients infected with hepatitis B virus show rapid progression of HIV infection clinical signs and are at a higher risk of liver cell damage compared to HIV mono-infection due to combined antiretroviral therapy (cART) [6]. Similarly, HIV patients when exposed to HBV show rapid progression of liver inflammation including cirrhosis, fibrosis and liver cancer compared to HBV mono-infection [8].

The impact of the HIV–HBV co-infection during pregnancy on mother’s health and the fetal pre-natal as well as post-natal life, mother-to-child-transmission (MTCT) of either virus or both is not fully understood [7, 9, 10]. However, the HIV–HBV co-infection during pregnancy impairs CD8+ T—cell and CD4+ T—cell specific HBV responses compared to mono-infection with HBV [11] exacerbating development of chronic HBV disease in the HBV–HIV co-infected [12]. Moreover, HBV–HIV co-infection has been implicated in accelerated hepatic apoptosis and fibrosis [13] increasing morbidity and mortality [6]. The effects of maternal HBV–HIV co-infection on the new born are diverse. Firstly, HBV infected children born to HBV–HIV co-infected mothers are at an increased risk of liver complications and liver diseases related death than those born from HBV mono-infected mothers [14]. Secondly, HBV–HIV co-infection escalates HBV replication and hepatitis B pre-core antigen (HBeAg) sero-positivity [6] increasing viral load augmenting the risk of MTCT of HBV [15, 16]. Moreover, the risk of mother-to-child transmission is 70–90% for HBeAg positive mothers compared to 10–40% for HBeAg negative mothers [17]*.* Unfortunately, 90–95% of the children who acquire HBV through the perinatal route progress to chronic infection later in life with increased risk of development of end stage liver diseases [16]. Therefore, understanding the HBV–HIV co-infection prevalence during pregnancy in our region and subsequent initiation of strategies to prevent MTCT of HBV should be of urgent attention by all health systems in SSA countries.

Whereas in most SSA countries HIV is routinely tested for in pregnant women attending antenatal care (ANC), HBsAg screening in the same cohort is not routine despite their overlapping routes of transmission [18–20]. This has hampered the design of interventions to mitigate the effect of HBV–HIV co-infection in pregnancy since the epidemiological data on the HBV–HIV co-infection among pregnant woman in SSA is scanty [21].

Failure to prevent MTCT of HBV from risky HBV–HIV co-infected pregnant mothers will culminate into a pool of HBV perinatal acquired infected children who will be a pool of continuous transmission to others. Thus information on the sero-prevalence of HBV–HIV co-infection among pregnant women is SSA is absolutely necessary for effective implementation of the control strategies.

The major aim of our meta-analysis was to estimate the prevalence of HBV–HIV co-infection among pregnant mothers in SSA following the common notion that most of HBV infections are co-infected with HIV due to the shared routes of transmission of the viruses. So, the study compared the prevalence of HBV–HIV co-infection among pregnant women on ANC in SSA with the prevalence reported in studies carried out elsewhere in the same cohort in order to assess the burden of the infection in our region and inform health care professionals, researchers and policy makers on the status quo of the HBV–HIV co-infection in this risky group of pregnant mothers. The SSA region has four sub-regions of East, West, Central and Southern Africa [22]. In East Africa (Ethiopia, Malawi, Rwanda, Tanzania and Uganda), in West Africa (Burkina Faso, Ivory coast, Ghana, Mali and Nigeria), in Central Africa (Cameroon and Democratic Republic of Congo) and in Southern Africa only South Africa had representative studies on the HBV–HIV co-infection in the data base for inclusion in our meta-analysis.

## Materials and methods

### Journal article search strategy

**Fig. 1:**
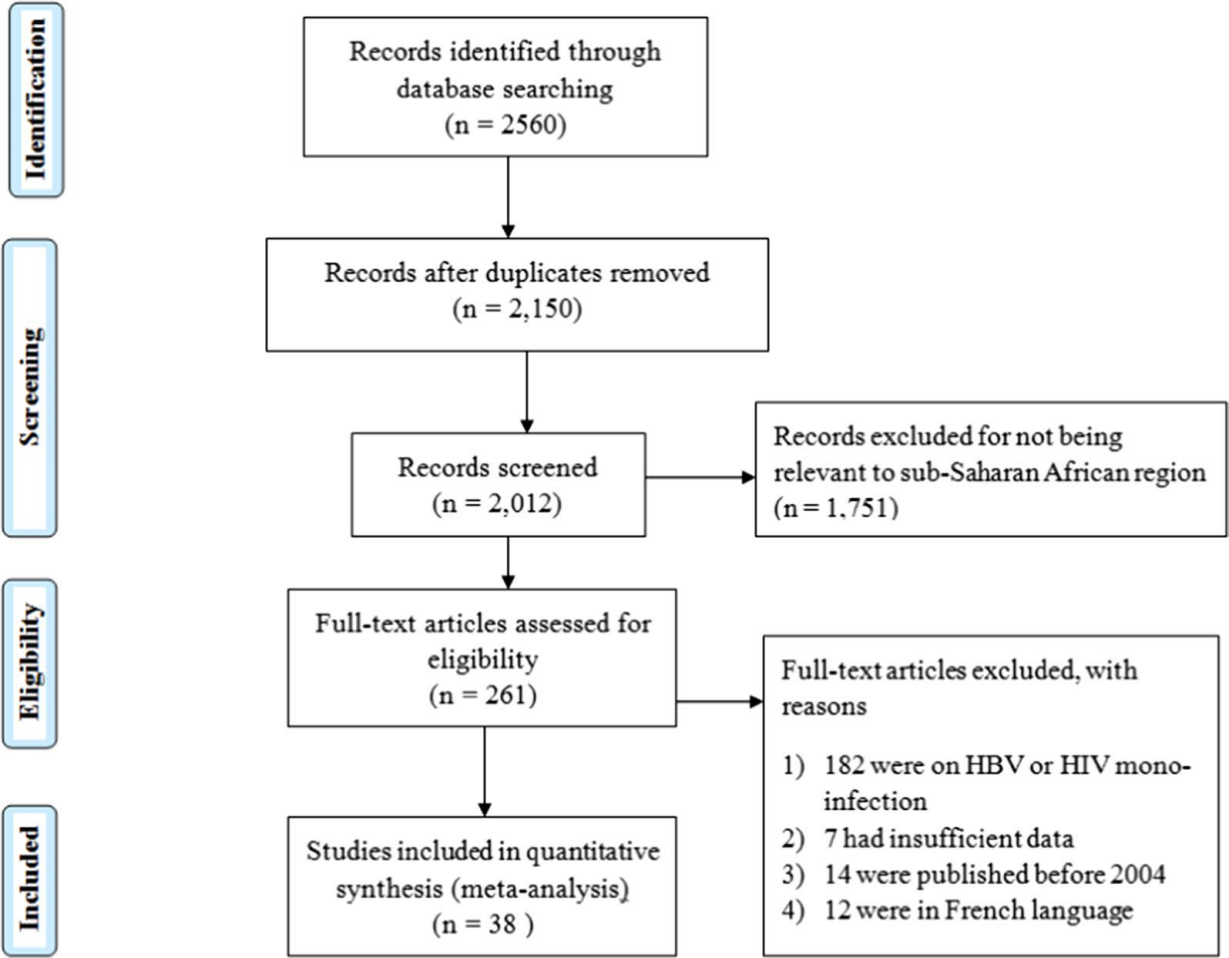
Flow chart for study eligibility following PRISMA criterion

This study was done in tandem with the Preferred Reporting Items for Systematic Reviews and Meta-analyses (PRISMA) standards (Fig. 1). Relevant studies were identified by a search of PubMed, Google scholar and ScienceDirect, as well as manual search in the references of the studies identified. The search was conducted from March 2020 to June 2020 using the following Boolean search terms: HBV–HIV co-infection in East Africa, West African, Central Africa, South Africa, Benin, Burkina Faso, Cape Verde, Côte D'Ivoire, Gambia, Ghana, Guinea, Guinea-Bissau, Liberia, Mali, Mauritania, Niger, Nigeria, Saint Helena, Senegal, Sierra Leone, Togo, Burundi, Comoros, Djibouti, Ethiopia, Eritrea, Kenya, Madagascar, Malawi, Mauritius, Mozambique, Réunion, Rwanda, Seychelles, Somalia, Somaliland, Tanzania, Uganda, Zambia, and Zimbabwe*,* Cameroon, Central African Republic, Chad, Congo Republic—Brazzaville, Democratic Republic of Congo, Equatorial Guinea, Gabon, São Tomé & Principe, Zambia, Zimbabwe, Botswana, Namibia, HIV positive, HIV–HBV co-infection, Antenatal care and pregnant women. The search results yielded about 2560 journal articles.

### Selection of articles for meta-analysis

The articles obtained were evaluated by two independent hepatitis B virus experts (HMK and DN) for their eligibility for inclusion in the study or exclusion from the study in line with the aim of the study.

### Inclusion criteria

The papers included in the study were selected after meeting the following preconditions; must have been a case–control or cohort study, testing for HBsAg, full text articles, from sub-Saharan Africa and published in peer-reviewed journals between the period of 2004 to 2019 in English language with sample size > 20 targeting HBV–HIV co-infected pregnant women on antenatal care in the general population or in the HIV positive cohort.

### Exclusion criteria

Articles excluded from the study included pre-prints, articles with sample size < 20, those with insufficient data, the studies whose main data could not be obtained, those investigating other viral hepatitis (C, D or E), reviews/meta-analyses, studies that detected hepatitis B core antigen (HBcAg), studies in other languages other than English, those published before 2004 and after 2019, studies targeting HBV mono-infection or HIV mono-infection or those conducted outside sub-Saharan Africa.

### Extracting data from the journal articles

Three of the authors (HMK, AW and DN) designed a protocol for the selection criteria aforementioned above. Both reviewers extracted data independently and entered the data in the spread sheet pending analysis. The two authors compared their records after the review of the journal articles and any differences were resolved by consensus. In our meta-analysis, the following characteristics were recorded for each study; first author, year of publication, country, study design, sampling technique, sample size, HBV–HIV co-infection and quality score.

### Quality assessment

The quality of each study was assessed by three independent reviewers (HMK, AW and DN) and the Newcastle–Ottawa scale was used [23]. Two authors (PO and HS) supervised the work of the HMK, AW and DN to ensure consistence in the quality of the work assessed. Three dimensions of comparability, selection and exposure were considered as described in the Newcastle–Ottawa scale. Studies were assigned scores ranging from the worst of zero to the best of 9. Studies with scores 9–8 were considered high quality studies, those between 7 and 6 were considered satisfactory whereas those with scores ≤ 5 were unsatisfactory and were rejected.

### Data analysis

Cochran’s Q test and I^2^ statistics were performed using the commercially available software (https://www.medcalc.org) to evaluate the extent of heterogeneity of all the eligible studies for meta-analysis. There was high heterogeneity among the pooled studies (I^2^ ≥ 78%) and the random effect model was used to pool the prevalence [24–26]. The prevalence for each study and 95% CI were calculated and the pooled prevalence estimate was determined. Some representative results were presented graphically using forest plots. The prevalence of each study in the forest plot is indicated by the purple circle/square. The size of the circle/square represents the weight contributed by each study in the meta-analysis. The pooled prevalence for random effect model is shown by the red diamond. The publication bias was assessed by carrying out funnel plots. Sources of heterogeneity were analyzed through sensitivity analysis, meta-regression and sub-group analysis. All analyses were performed using the statistical software, MedCalc available at https://www.medcalc.org

## Results

The PRISMA strategy (Fig. 1) was used to screen for the eligible studies. In total, 38 (thirty-eight) articles with a total sample size of 44,114 (forty-four thousand one hundred fourteen) pregnant women attending antenatal care and 1047 (one thousand forty-seven) HBV–HIV co-infected pregnant mothers.

In our meta-analysis, 13(thirteen) eligible studies were from East African region [27–38], sixteen (16) studies were from West African region [18, 39–53], 2 (two) studies were from South African region [14, 54] and 7 (seven) studies were from Central African region [55–61]. In East Africa, five (5) studies were from Ethiopia [27–31], 2 (two) from Malawi [32, 33], 2 (two) from Rwanda [34, 35], 1 (one) from Tanzania [36] and 3 (three) from Uganda [35, 37, 38]. In West Africa, 2 (two) studies were from Burkina Faso [39, 40], 3 (three) from Ghana [41–43], 1 (one) from Ivory Coast [44], 1 (one) from Mali [45] and 9 (nine) from Nigeria [18, 46–53]. In southern Africa region, both studies were from South Africa [14, 54]. Finally, in Central Africa, 6 (six) studies were from Cameroon [55–60] and 1(one) eligible study was from Democratic republic of Congo [61] (Table 1).

**Table 1:** Characteristics of the eligible studies

First author, year	Country	Study design	Sampling technique	Sample	HBV/HIV co-infection	Diagnostic Method	QS
Andersson et al. [14]	South Africa	Cross-section	Purposive	3089	53	ELISA	8
Bafa et al. [27]	Ethiopia	Cross-section	Random	222	4	RDT	9
Chasela et al. [32]	Malawi	Cross-section	Entire	2048	103	ELISA	9
Desalegn et al. [28]	Ethiopia	Cross-section	Entire	202	1	EIA	6
Dionne-Odom et al. [60]	Cameroon	Cross-section	Consecutive	7069	205	RDT	9
Ezechi et al. [46]	Nigeria	Cross-section	Purposive	2391	101	ELISA	9
Ilboudo et al. [39]	Burkina Faso	Cross-section	Purposive	115	14	RDT	6
Mbaawuaga et al. [47]	Nigeria	Cross-section	Purposive	507	5	RDT	7
Mpody et al. [61]	DR Congo	Cross-section	Entire	1377	65	ELISA	8
Abwonga et al. [55]	Cameroon	Cross-section	Entire	301	5	RDT	7
Adesina et al. [48]	Nigeria	Cross-section	Entire	721	64	ELISA	7
Amsalu et al. [29]	Ethiopia	Cross-section	Purposive	475	10	RDT	8
Andreotti et al. [33]	Malawi	Cross-section	Entire	309	28	RDT	7
Bassey et al. [49]	Nigeria	Cross-section	Purposive	500	36	ELISA	8
Bayo et al. [37]	Uganda	Cross-section	Random	402	4	ELISA	7
Dabsu et al. [30]	Ethiopia	Cross-section	Convenient	421	1	RDT	8
Fomulu et al. [56]	Cameroon	Cross-section	Consecutive	959	7	ELISA	8
Frempong et al. [41]	Ghana	Cross-section	Consecutive	248	22	EIA	8
Helegbe et al. [42]	Ghana	Cross-section	Purposive	3127	1	VCIA	9
Ikeako et al. [50]	Nigeria	Retrospective	Entire	1239	3	ELISA	9
Kfutwah et al. [57]	Cameroon	Cross-section	Entire	650	28	RDT	8
Lar et al. [18]	Nigeria	Cross-section	Entire	135	16	ELISA	8
MacLean et al. [45]	Mali	Cross-section	Entire	3659	14	ELISA	8
Manyahi et al. [36]	Tanzania	Cross-section	Consecutive	249	7	ELISA	8
Mutagoma et al. [34]	Rwanda	Cross-section	Entire	13,121	20	ELISA	9
Noubiap et al. [58]	Cameroon	Cross-section	Consecutive	325	5	RDT	7
Ntiamoah et al. [43]	Ghana	Cross-section	Purposive	124	11	RDT	6
Ojiegbe et al. [51]	Nigeria	Cross-section	Purposive	300	3	ELISA	6
Pirillo et al. [35]	Rwanda	Cross-section	Entire	82	2	RDT	7
Pirillo et al. [35]	Uganda	Cross-section	Entire	164	8	ELISA	7
Rouet et al. [44]	Ivory cost	Retrospective	Entire	449	45	ELISA	8
Seremba et al. [38]	Uganda	Cross-section	Purposive	612	9	AAS	7
Simpore et al. [40]	Burkina Faso	Cross-section	Entire	336	24	AAS	7
Tanjong Re et al. [59]	Cameroon	Cross-section	Purposive	406	6	AAS	8
Thumbiran et al. [54]	South Africa	Cross-section	Entire	570	18	ELISA	6
Usanga et al. [52]	Nigeria	Cross-section	Purposive	562	5	ELISA	8
Ya’aba et al. [53]	Nigeria	Cross-section	Purposive	330	90	ELISA	7
Zenebe et al. [31]	Ethiopia	Cross-section	Random	318	4	RDT	9

*VCIA* vitros chemi luminescent immunoassay, *AAS* abbott ARCHITECT system, *ELISA* enzyme linked immuno assay, *EIA* enzyme immuno assay, *RDT* rapid diagnostic test, *QS* quality score

Majority of the studies included in our meta-analysis (30/38) or 78.95% were published from 2011 to 2019 [14, 18, 27–34, 36–38, 41–43, 45–47, 50, 51, 53–61] and a few studies (8/38) or 21.05% were published from 2004 to 2010 [35, 39, 40, 44, 48, 49, 52]. Additionally, most studies; 27/38 (71%) were conducted on pregnant mothers on ANC from the general population [14, 27–31, 34–42, 45, 47–52, 54, 56, 58–60] whereas a few of the studies;11/38 (29%) used HIV positive mothers to survey for the prevalence of HBV–HIV co-infection among ANC attendees in SSA [18, 32, 33, 43, 44, 46, 53, 55, 57, 61].

Finally, 19 (nineteen) of the eligible studies detected the HBsAg using Enzyme Linked Immunosorbent Assay (ELISA) [26, 27, 31, 33, 35, 37, 39, 41, 43, 46–50, 52, 54, 56, 59], 13 (thirteen) used Rapid Diagnostic Techniques (RDT) [18, 29, 30, 38, 40, 42, 45, 51, 53, 55, 58, 60, 61] and 3 (three) used Abbott ARCHITECT system (AAS) [38, 40, 59], 1 (one) Vitros Chemiluminescence Immunoassay (VCIA) [42] and 2 (two) Enzyme Immuno Assay (EIA) [28, 41].

### Prevalence of HBV–HIV co-infection among pregnant mothers in Sub-Saharan African region

The prevalence of HBV–HIV co-infection in Sub-Saharan African region varied widely with the highest prevalence reported among HIV positive pregnant mothers on ANC compared to pregnant mothers on ANC from the general population. Among the HIV positive mothers, the HBV–HIV co-infection prevalence ranged from 1.661% (95% CI = 0.542 to 3.834%) in 301(three hundred one) Cameroonian mothers on ANC [55] to 27.273% (95% CI = 22.593 to 32.420%) in a sample of 330 (three hundred thirty) Nigerian pregnant mothers [53]. On the other hand, HBV–HIV co-infection prevalence among mothers from the general population ranged from 0.03% (95% CI = 0.00081 to 0.178%) in a sample of 3,127 (three thousand one hundred twenty-seven) Ghanaian mothers on ANC [42] to 12.174% (95% CI = 6.818 to 19.582%) in a sample of 115 (one hundred fifteen) Burkinabé mothers on ANC [39].

**Fig. 2:**
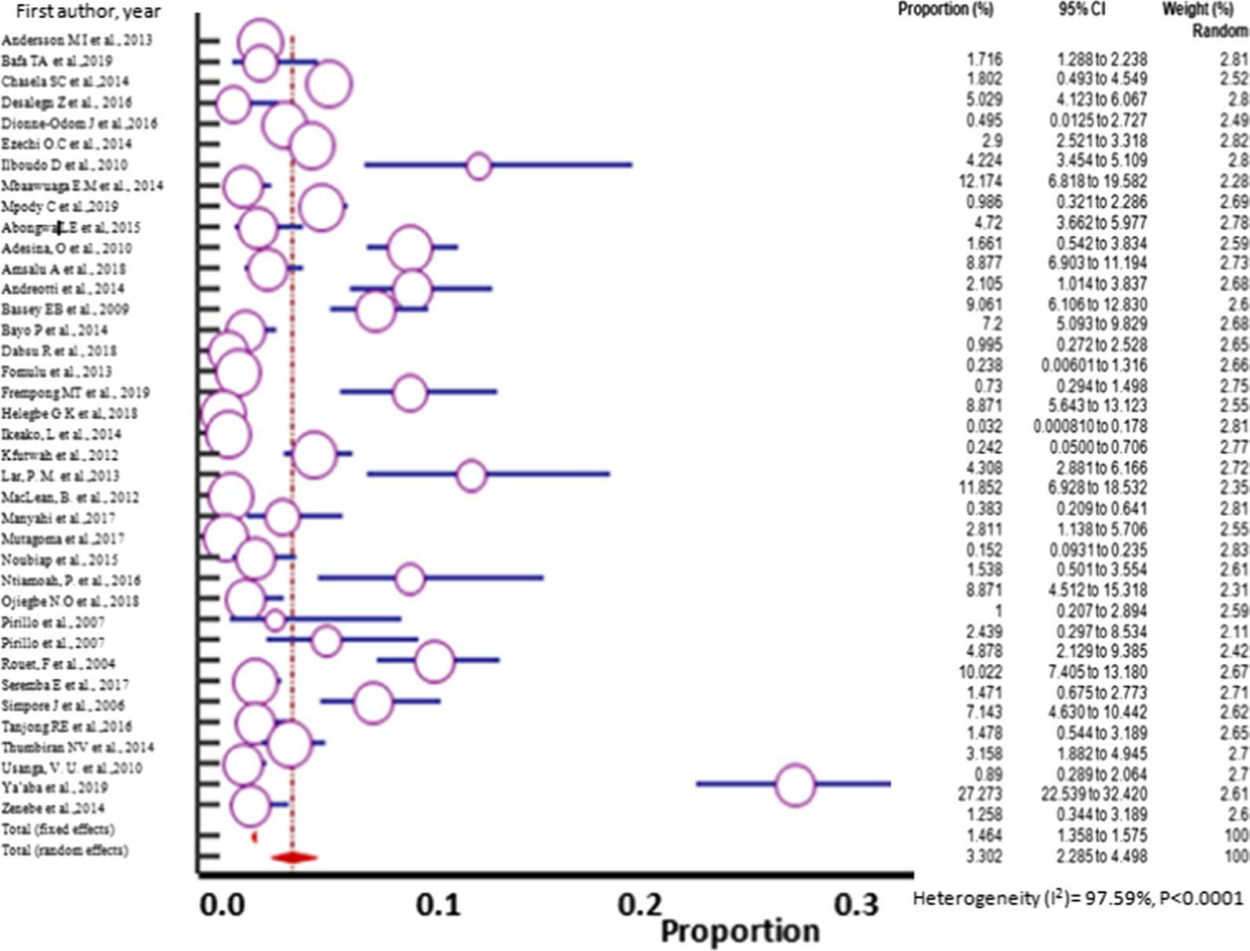
Forest plot for the Pooled prevalence estimate of HBV–HIV co-infection among pregnant mothers on antenatal care in Sub-Saharan Africa from 2004–2019 by random effect model

However, the overall pooled prevalence of HBV–HIV co-infected was 3.303% (95% CI = 2.285 to 4.498%) among the sample of 44,114 (forty-four thousand one hundred fourteen) with heterogeneities (I^2^) of 97.59% (*P* > 0.0001) (Fig. 2).

**Fig. 3:**
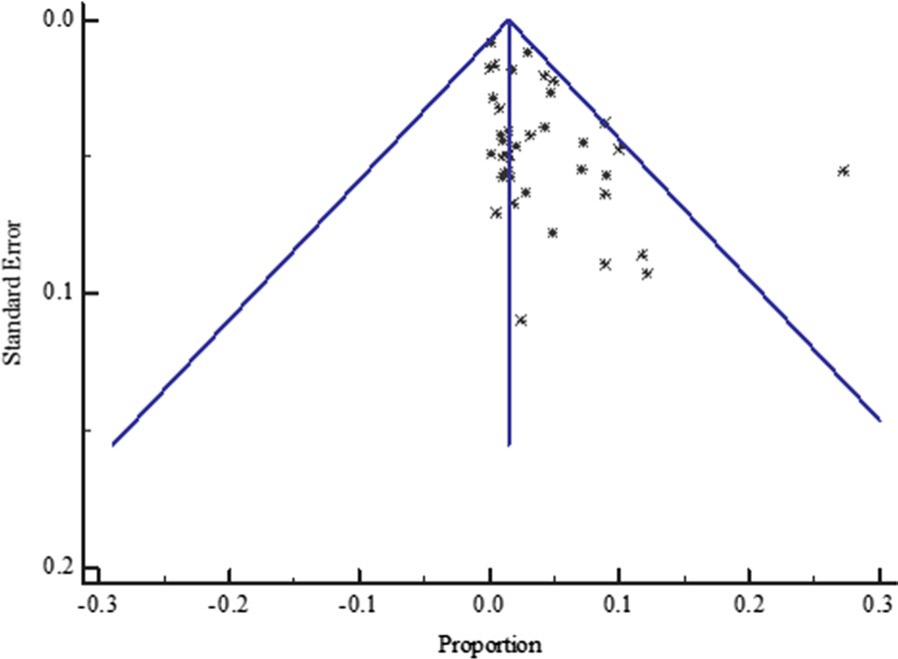
Bias assessment funnel plot for HBV–HIV co-infection prevalence among pregnant mothers on antenatal care in Sub-Saharan Africa 2004 to 2019

The funnel plot displayed a symmetric spread of studies in terms of relative weight and effect size despite the significant heterogeneity (*P* < 0.0001), thereby indicating little evidence of publication bias (Fig. 3).

### Sub-group meta-analysis

We sub divided our meta-analysis into groups which included sub-Saharan African regions, year of publication, HIV + cohort or not and HBsAg diagnostic method (Table.2). In all sub-group meta-analyses, the heterogeneity remained high (I^2^ > 89%, *P* < 0.0001) except for South Africa with a lowered heterogeneity (I^2^ = 78.40%, *P* = 0.00314). By region, the highest and the lowest pooled prevalence estimate of HBV–HIV co-infection among pregnant women attending antenatal care were registered from West Africa, 5.155% (95%CI = 2.671 to 8.392%) in a sample of 14,743 (fourteen thousand seven hundred forty-three) and East Africa, 2.168% (0.830 to 4.116%) in a sample of 18,625 (eighteen thousand six hundred twenty-five) respectively.

**Table 2:** Sub-group meta-analysis of the HBV–HIV co-infection pooled prevalence estimation among ANC attendees

Variable	Analysis				
	number	Prevalence % (95% CI)	*P* value	I^2^ (%) (95% CI)	P _het_
Region					
West Africa	16	5.16 (2.67 to 8.39) REF		92.25 (97.82 to 98.6)	*P* < 0.0001
East Africa	13	2.168 (0.830 to 4.116)	< 0.001*	96.79(95.68 to 97.62)	*P* < 0.0001
South Africa	2	2.317 (1.105 to 3.959)	< 0.001*	78.4(6.03 to 95.03)	*P* = 0.0314
Central Africa	7	2.441 (1.492 to 3.617)	< 0.001*	88.02(77.70 to 93.57)	*P* < 0.0001
Year of publication					
2004–2010	8	6.356 (3.611 to 9.811)		91.15(84.97 to 94.79)	*P* < 0.0001
2011–2019	30	2.252 (1.452 to 3.221)	< 0.001*	97.15(96.56 to 97.64)	*P* < 0.0001
HIV + Cohort					
Yes	12	8.312 (5.806 to 11.22)		94.90(92.69 to 96.45)	*P* < 0.0001
No	27	2.152 (1.358 to 3.125)	< 0.001*	96.73(95.98 to 97.33)	*P* < 0.0001
Detection method					
ELISA	19	3.39 (2.06 to 5.047) REF		93.80(91.64 to 95.41)	*P* < 0.0001
RDT	13	3.167 (1.484 to 5.449)	0.2996	98.01(97.44 to 98.46)	*P* < 0.0001
Others	6	3.323 (0.955 to 7.050)	0.7443	98.89(98.46 to 99.20)	*P* < 0.0001

^*^P value is statistically significant, HIV = Human Immunodeficiency Virus, ELISA = Enzyme Linked Immuno Assay, RDT = Rapid Diagnostic Test

**Fig. 4:**
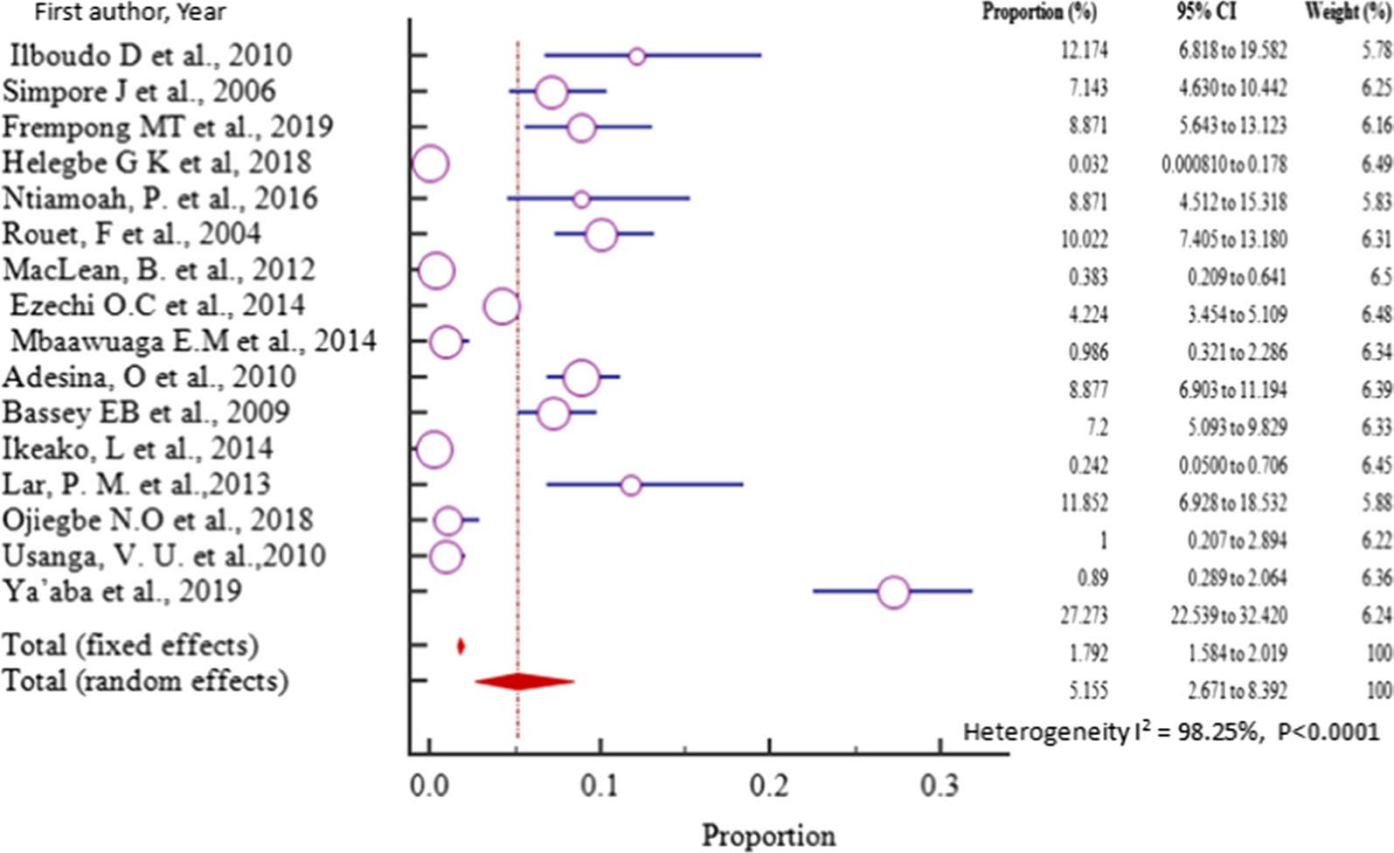
Forest plot for the Pooled prevalence estimate of HBV–HIV co-infection among pregnant mothers on antenatal care in West Africa from 2004–2019 by random effect model

The prevalence of HBV–HIV co-infection among pregnant women in West Africa varied from 0.0320% (95% CI = 0.00081 to 0.178%) reported in a study conducted in Ghana [42] to 27.273% (95% CI = 22.539 to 32.420%) reported in a study done in Nigeria [53] with pooled prevalence estimate of 5.155% (95% CI = 2.671 to 8.392%) in a sample of 14,743 (fourteen thousand seven hundred forty three) pregnant women attending antenatal care (Fig. 4, Table 2) and was significantly higher than any other region of sub-Saharan Africa (*P* < 0.001).

**Fig. 5:**
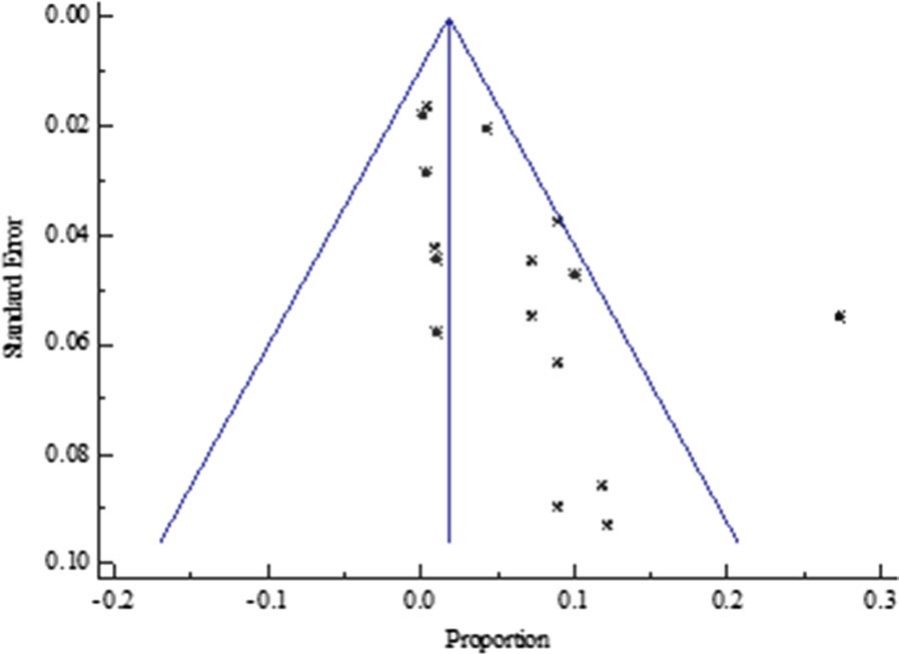
Bias assessment funnel plot for HBV–HIV co-infection prevalence rate among pregnant mothers on antenatal care in West Africa 2004 to 2019

The funnel plot for all the West African studies published from 2004 to 2019 displayed a symmetric spread of studies in terms of relative weight and effect size despite the significant heterogeneity, I^2^ = 98.25% (*P* < 0.0001), thereby indicating little evidence of publication bias (Fig. 5).

**Fig. 6:**
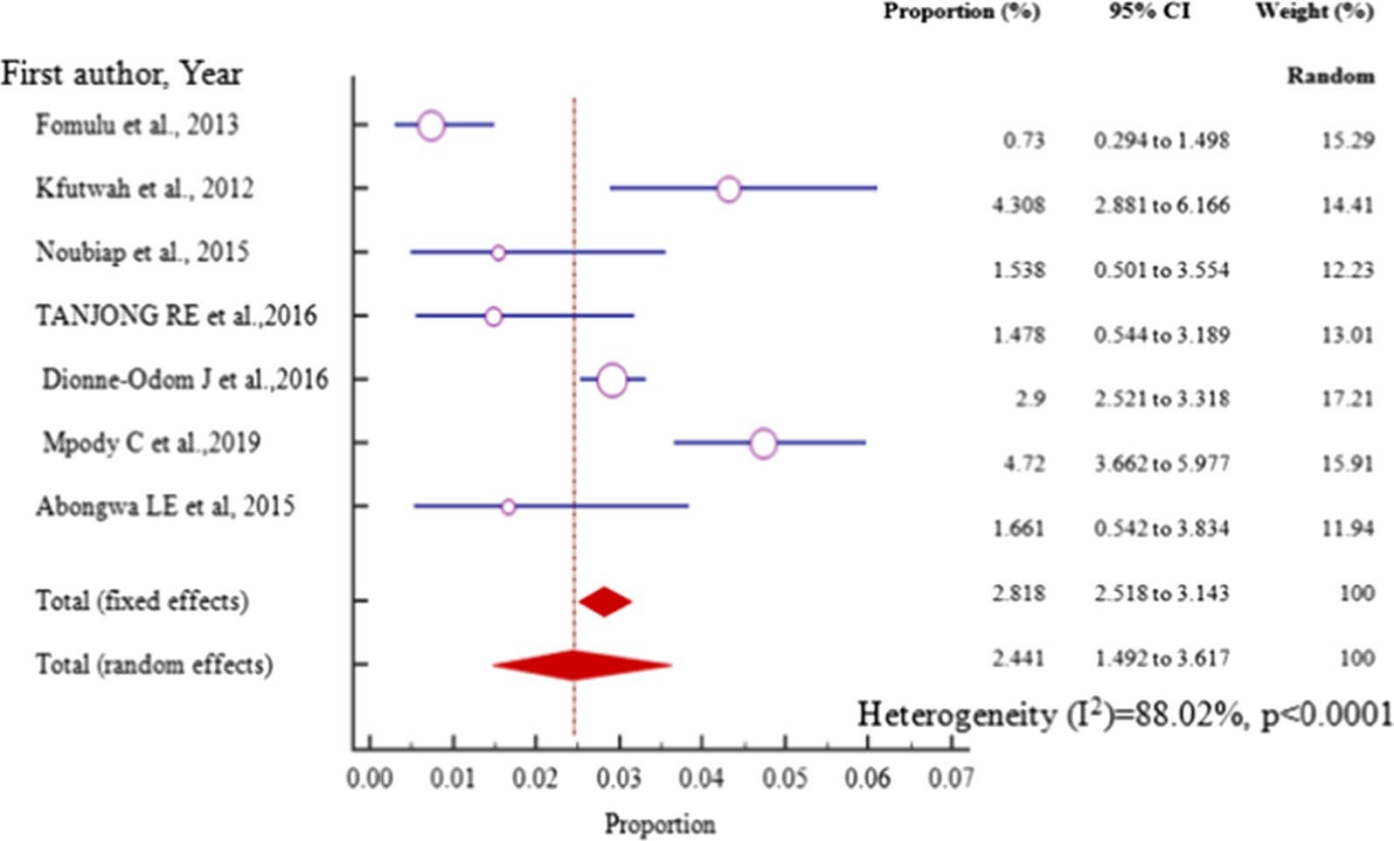
Forest plot for the Pooled prevalence estimate of HBV–HIV among pregnant mothers on antenatal care in Central Africa from 2012–2019 by random effect model

The prevalence of HBV–HIV co-infection among pregnant women on ANC in Central Africa varied from 0.73% (95% CI = 0.294 to 1.498%) reported in a study conducted in Cameroon [56] to 4.72% (95% CI = 3.662 to 5.977%) reported in a study done in Democratic Republic of Congo (DRC) [61] with pooled prevalence estimate of 2.441%(95% CI = 1.492 to 3.617%) in a sample of 11,087 (eleven thousand eighty-seven) pregnant women attending antenatal care (Fig. 6, Table 2).

**Fig. 7:**
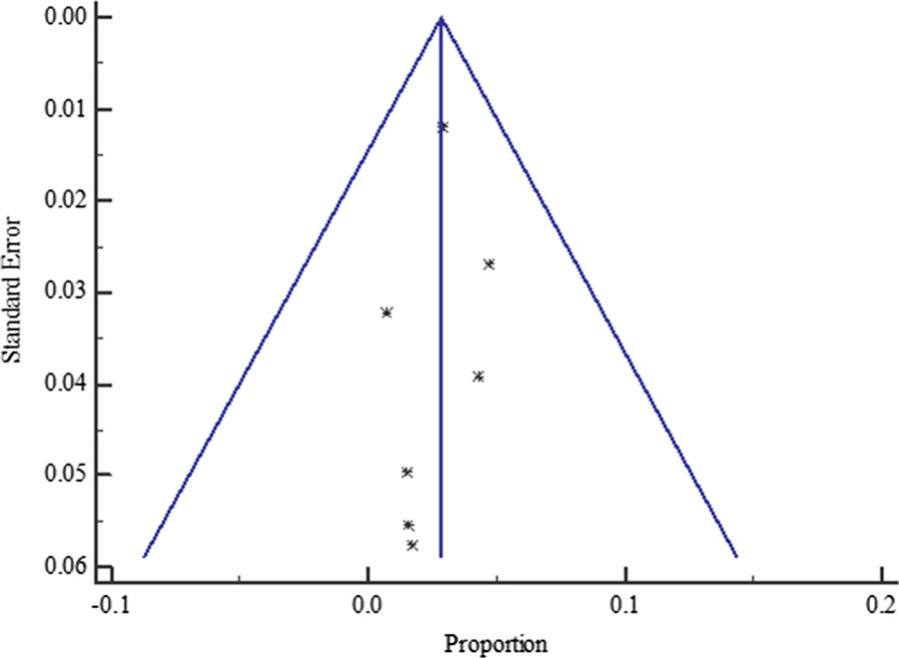
Bias assessment funnel plot for HVB-HIV co-infection prevalence rate among pregnant mothers on antenatal care in Central Africa 2012 to 2019

Similarly, the funnel plot for all the Central African studies published between 2012 to 2019 displayed a symmetric spread of studies in terms of relative weight and effect size despite the significant heterogeneity, I^2^ = 88.02% (*P* < 0.0001), thereby indicating little evidence of publication bias (Fig. 7).

**Fig. 8:**
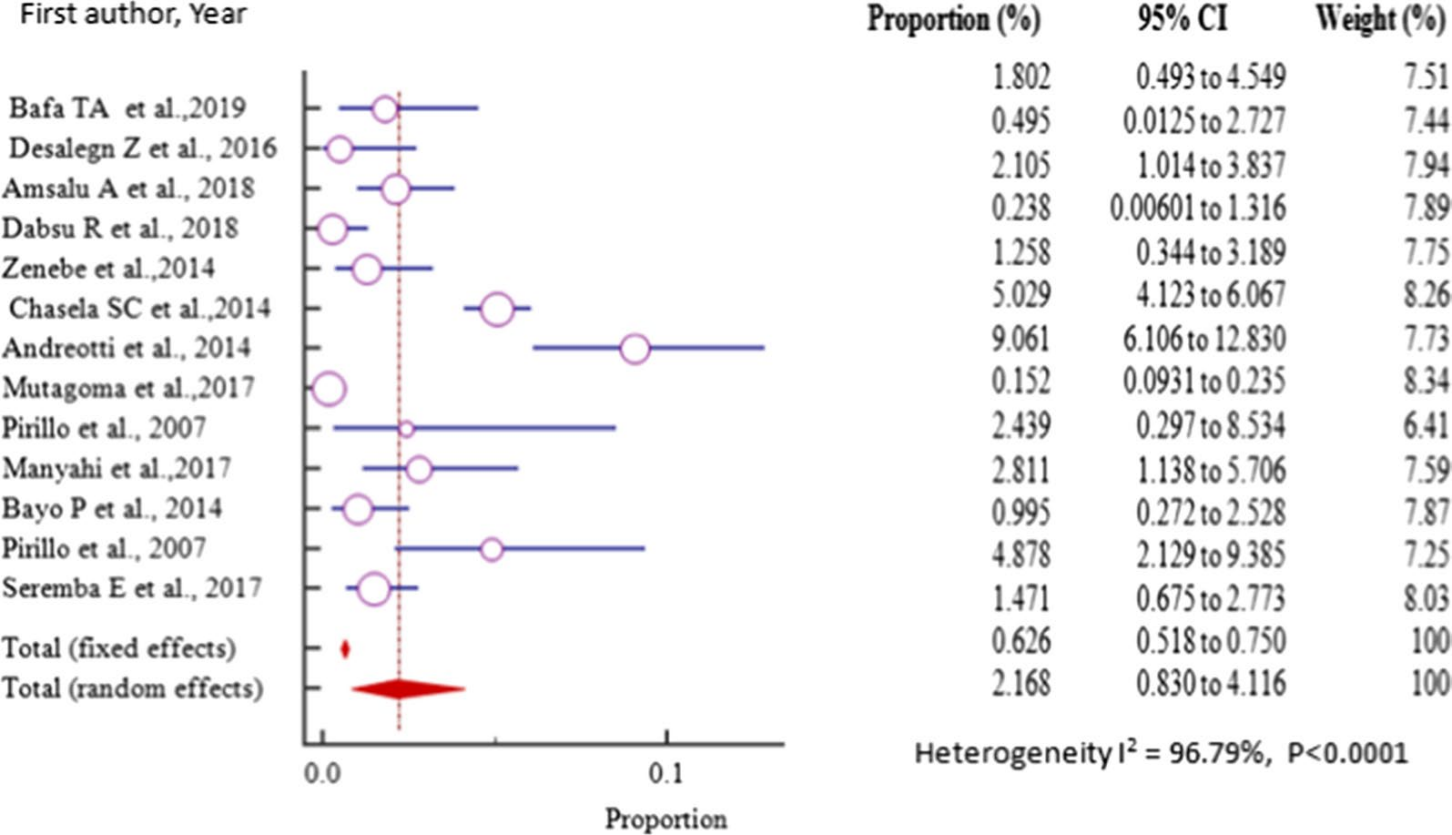
Forest plot for the Pooled prevalence estimate of HBV–HIV co-infection among pregnant mothers on antenatal care in East Africa from 2007–2019 by random effect model

The prevalence of HBV–HIV co-infection among pregnant women in East Africa varied from 0.152%(95% CI = 0.0931 to 0.235%) reported in a study conducted in Rwanda from the general population [34] to 4.878% (95% CI = 2.129 to 9.385%) in a related study conducted in Uganda [35] with pooled prevalence estimate of 2.168% (95% CI = 0.830 to 4.116%) in a sample of 18,625 (eighteen thousand six hundred twenty-five) pregnant women attending antenatal care (Fig. 8, Table 2).

**Fig. 9:**
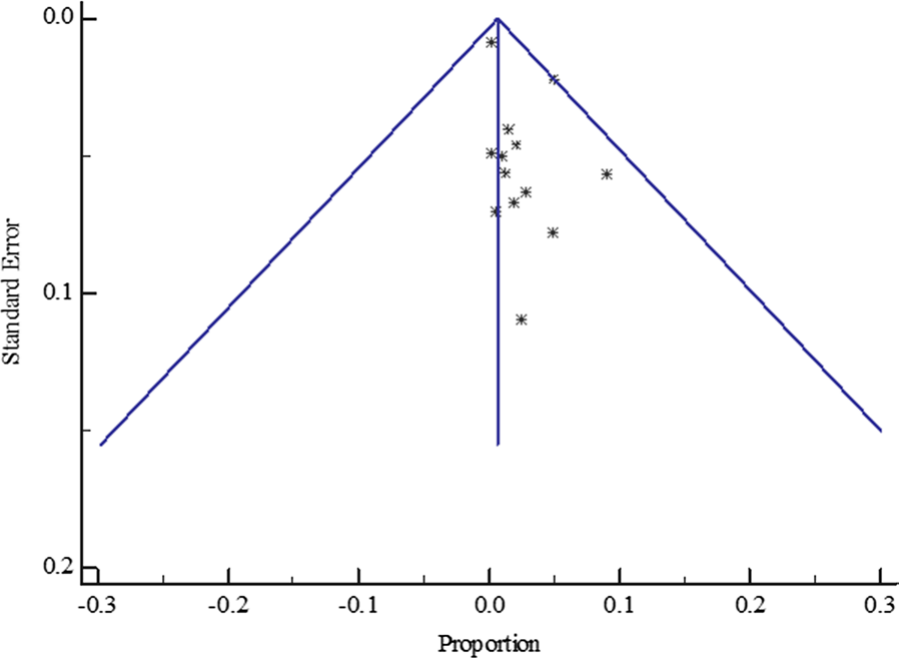
Bias assessment funnel plot for HBV–HIV co-infection prevalence rate among pregnant mothers on antenatal care in East Africa 2007 to 2019

Again, the funnel plot for all the East African studies published from 2007 to 2019 displayed a symmetric spread of studies in terms of relative weight and effect size despite the significant heterogeneity, I^2^ = 96.79% (*P* < 0.0001), thereby indicating little evidence of publication bias (Fig. 9).

**Fig. 10:**
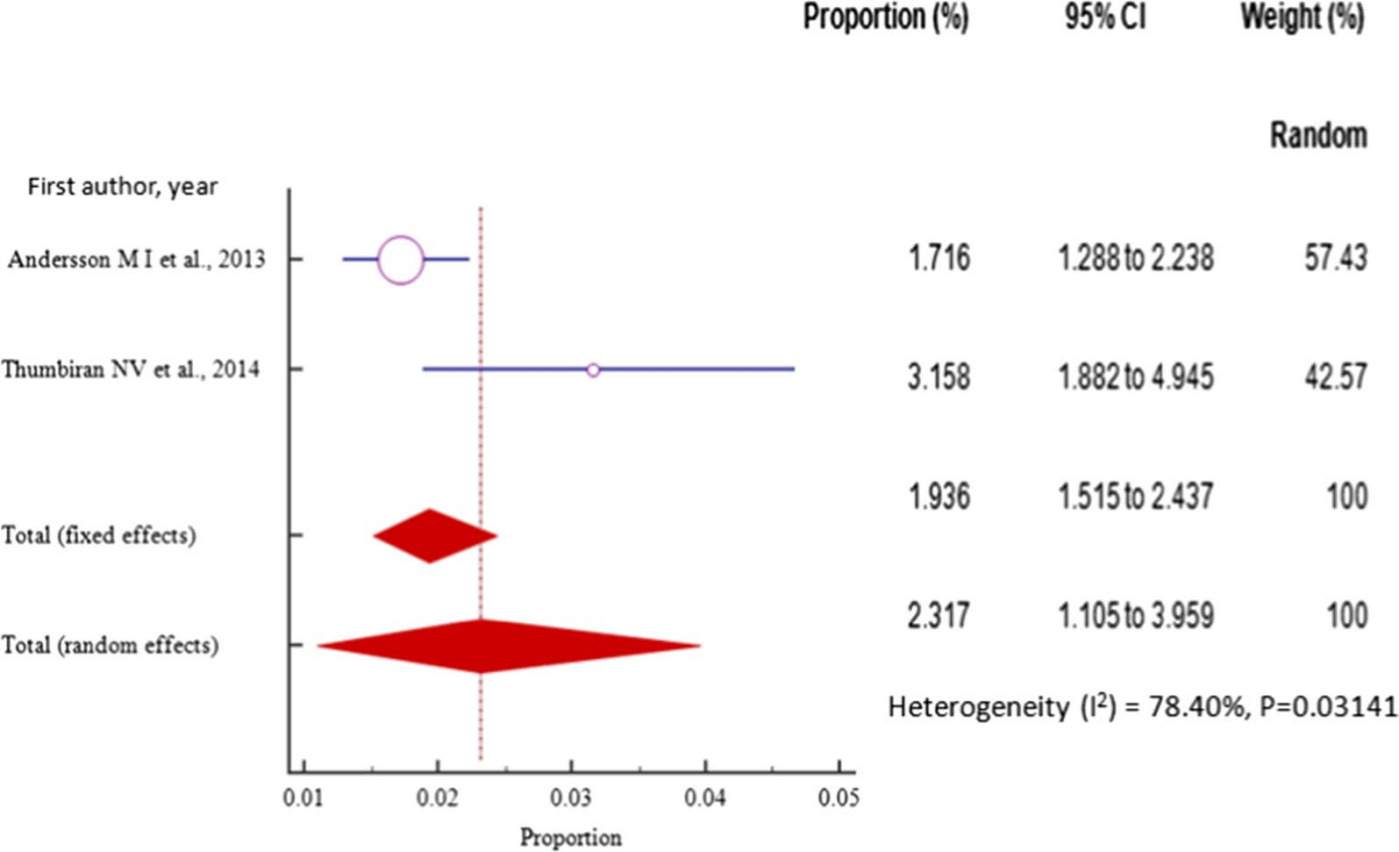
Forest plot for the Pooled prevalence estimate of HBV–HIV co-infection among pregnant mothers on antenatal care in East Africa from 2012–2014 by random effects model

The prevalence of HBV–HIV co-infection among pregnant women in South Africa varied from 1.716% (95% CI = 1.288 to 2.238%) in a study conducted in south Africa from the general population [14] to 3.158% (95% CI = 1.882 to 4.945%) in a study done in the same country and cohort [54] with pooled prevalence estimate of 2.317% (95% CI = 1.105to 3.959%) in a sample of 3,659 (three thousand six hundred fifty-nine) pregnant women on ANC (Fig. 10, Table 2).

**Fig. 11:**
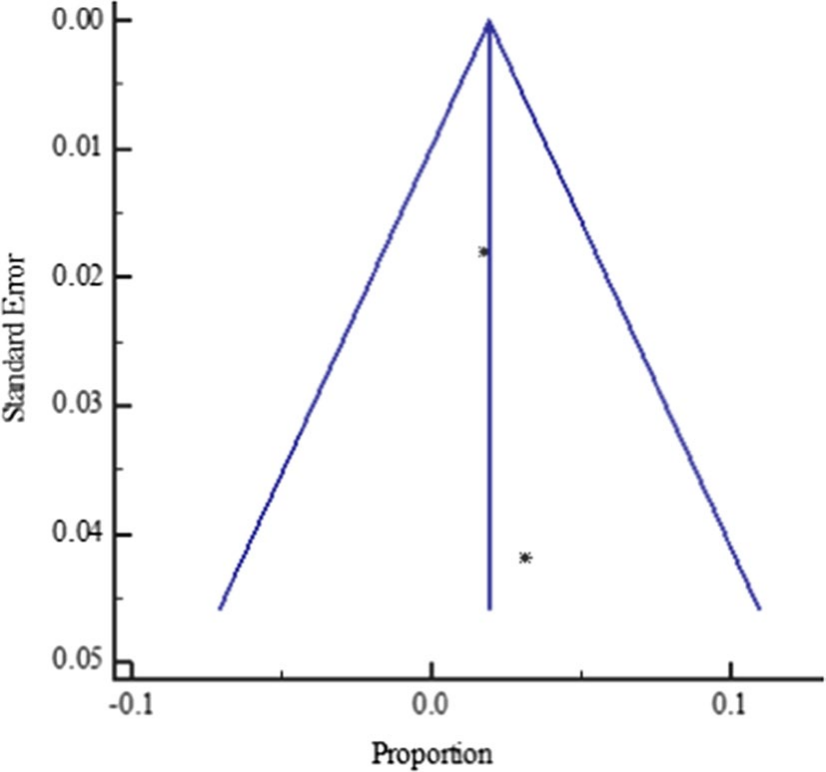
Bias assessment funnel plot for HBV–HIV co-infection prevalence rate among pregnant mothers on antenatal care in South Africa 2013 to 2014

As with the other regional subgroup meta-analyses, the funnel plot for the two South African studies published from 2013 to 2014 displayed a symmetric spread of studies in terms of relative weight and effect size despite the significant heterogeneity, I^2^ = 78.4% (*P* = 0.0314), thereby indicating little evidence of publication bias (Fig. 11).

Regarding the year of publication, the lowest prevalence of 0.032% (95% CI = 0.000810 to 0.178%) of HBV–HIV co-infection in Sub-Saharan African region was reported in the study published in 2018 conducted from Ghana on 3,127 (three thousand one hundred twenty seven) pregnant mothers attending antenatal care [42] whereas the highest prevalence of 27.273% (95% CI = 22.593 to 32.420%) was noted in a study published in 2019 conducted from Nigeria among 330 (three hundred thirty) HIV + pregnant mothers [53].

When we dichotomized the years of publication into 2004 to 2010 and 2011 to 2019, recently published studies (2011 to 2019) reported significantly lower pooled prevalence estimate of HBV–HIV co-infection, 2.252% (95% CI = 1.740 to 3.765%) in a sample of 45,185 (forty five thousand one hundred eighty five) pregnant women on antenatal care (*P* < 0.001)compared to earlier studies (2004–2010) with higher HBV–HIV pooled prevalence estimate of 6.356% in a sample of 2,929 (two thousand nine hundred twenty nine) pregnant women (95% CI = 3.611 to 9.811%).

When we meta-analyzed data on whether the studies used HIV positive cohort or not to assess the prevalence of HBV–HIV co-infection among the pregnant mothers, interesting results were obtained. The lowest and highest HBV–HIV co-infection prevalence estimates of 1.661% (95% CI = 0.542 to 3.834%) and 27.273% (95% CI = 22.539 to 32.420%) in a cohort of HIV positive mothers were reported in Cameroon [55] and in Nigeria [53] respectively. On the other hand, the lowest and highest HBV–HIV co-infection prevalence of 0.032% (95% CI = 0.000810 to 0.178%) and 12.174% (95% CI = 6.818 to 19.582%) in the general population of mothers on antenatal care were found in studies reported from Ghana [42] and Burkina Faso [39] respectively. Whereas all the eligible studies included in our meta-analysis focused on HBV–HIV co-infection either in the general population or in HIV positive cohort pregnant mothers on antenatal care, one study [14] investigated the HBV–HIV co-infection both in the general population and among the HIV positive cohort. Indeed, the HIV positive pregnant mothers had a significantly higher HBV–HIV co-infection pooled prevalence of 8.312% (95% CI = 5.806 to 11.220%) in a sample of 9,332 (nine thousand three hundred thirty-two) HIV positive cohort compared to the HBV–HIV co-infected mothers in the general population with a pooled prevalence estimate of 2.152%, (95% CI = 1.358 to 3.125%) (*P* < 0.001) in a sample of 39,388 (thirty-nine thousand three hundred eighty-eight) pregnant mothers. Finally, the prevalence of HBV–HIV co-infection prevalence in the separate studies appears not to have been confounded by the method used for the detection of the HBsAg since when we meta-analyzed data on the detection method of the HBsAg, the results did not differ significantly (*P* > 0.05).

### Risk factors for HBV–HIV co-infection

**Fig. 12:**
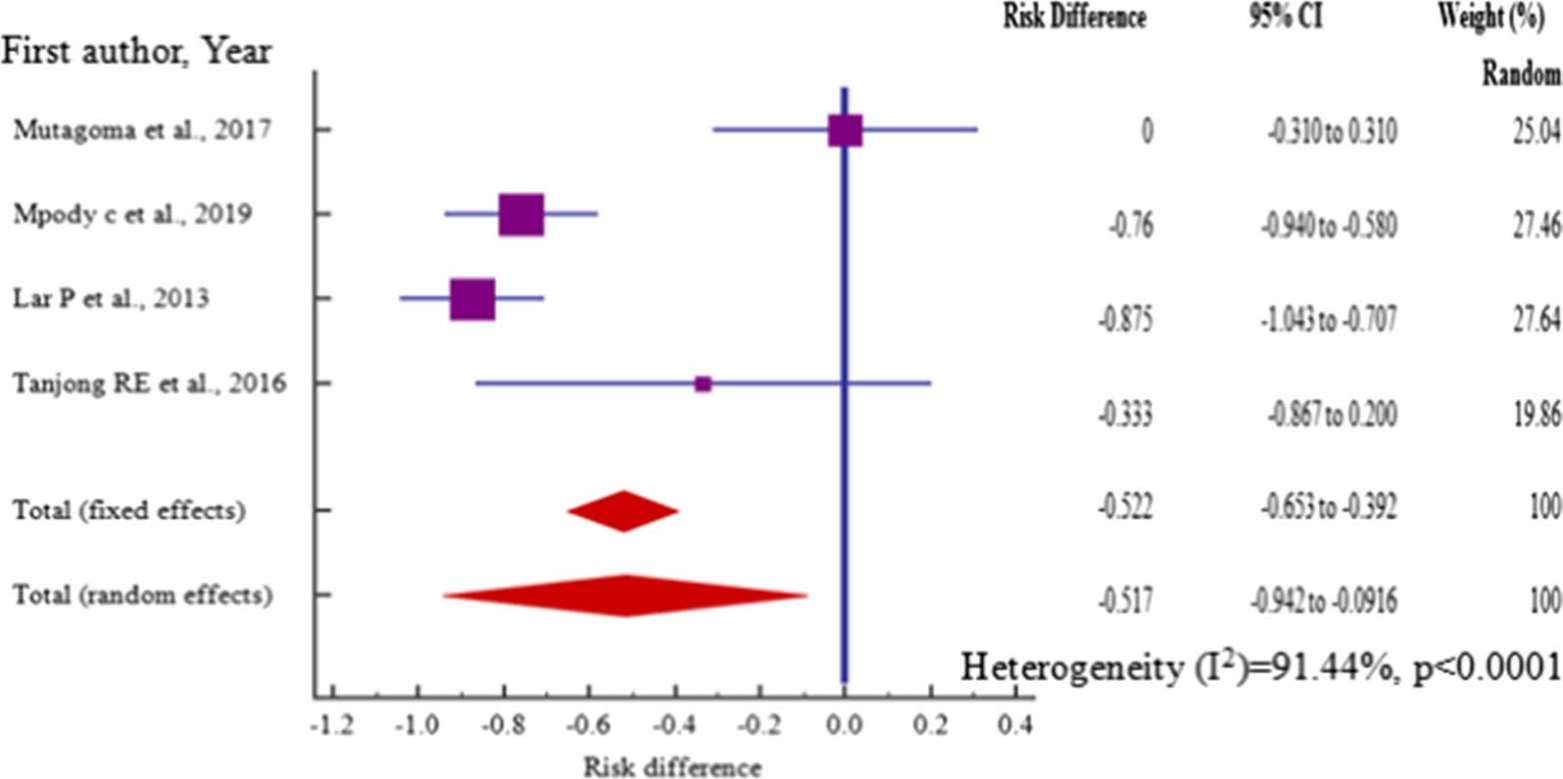
Forest plot of risk difference for HBV–HIV co-infection for pregnant mothers attending antenatal care < 25 years and > 25 years from cross-section/cohort studies in sub-Saharan Africa from 2014 to 2019

**Fig. 13:**
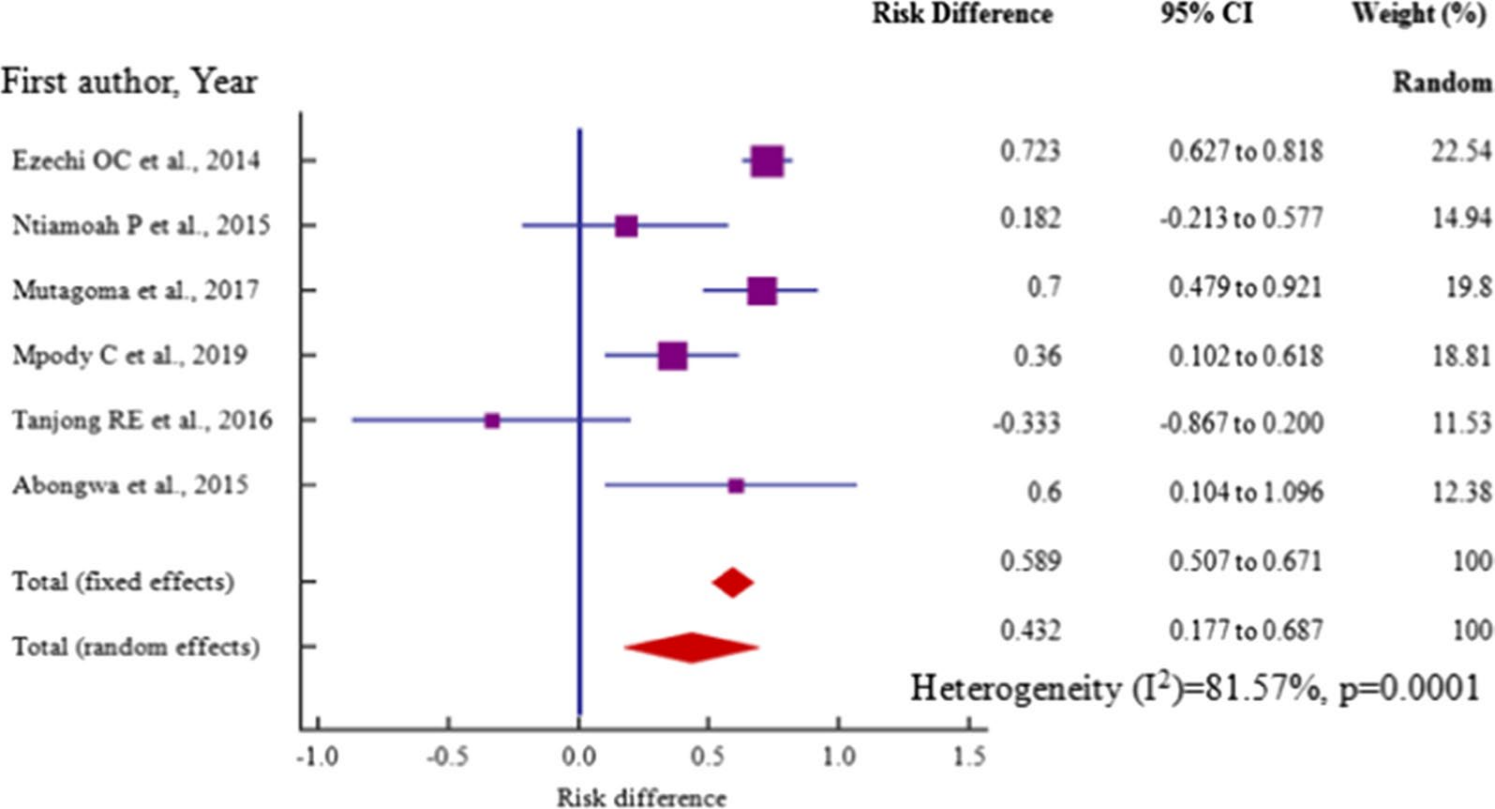
Forest plot of risk difference for HBV–HIV co-infection among married and unmarried pregnant mothers from cross-section/cohort studies in sub-Saharan Africa from 2014 to 2019

**Fig. 14:**
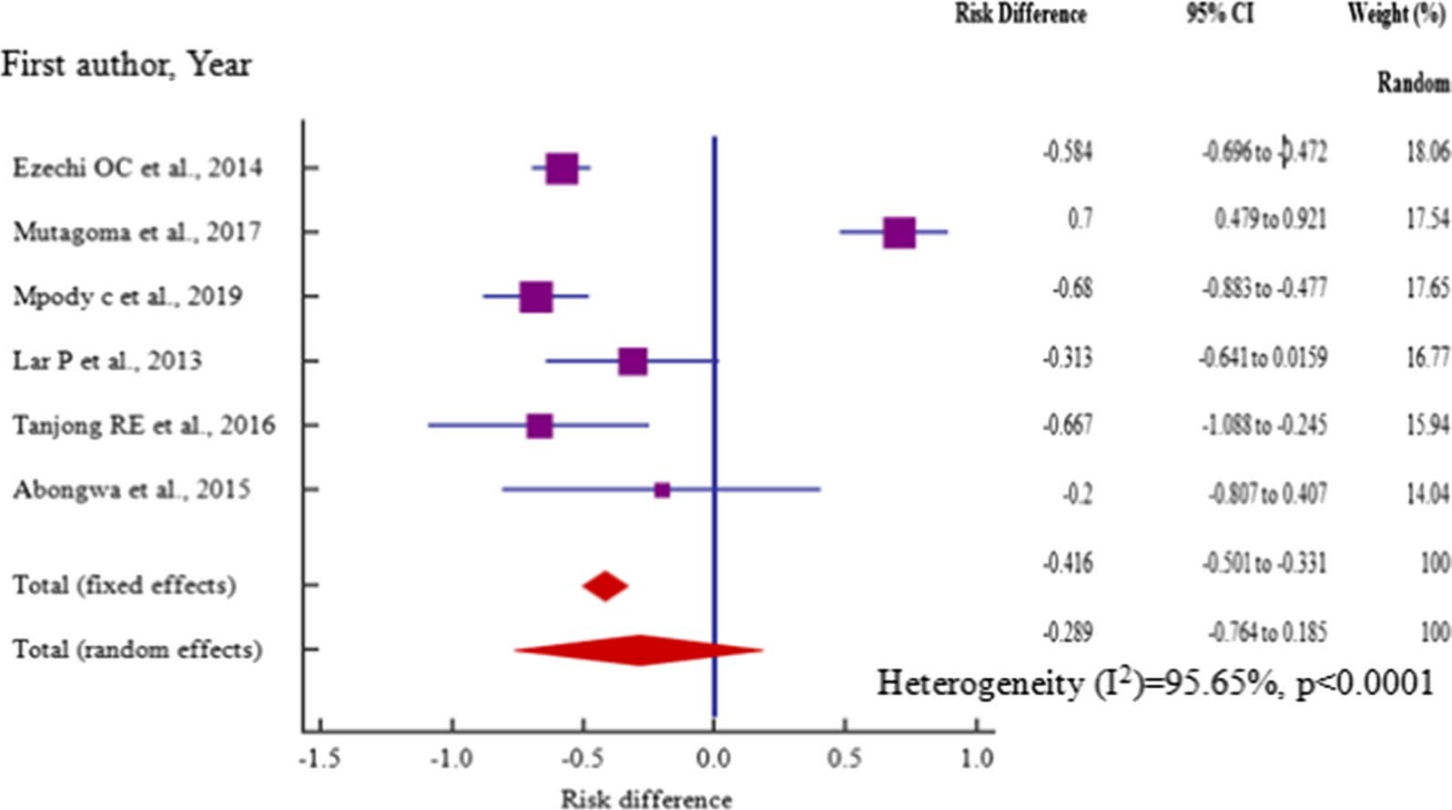
Forest plot of risk difference for HBV–HIV co-infection among educated and uneducated pregnant mothers from cross-section/cohort studies in sub-Saharan Africa from 2014 to 2019

Age, risk difference -0.517 (95% CI = −0.942 to −0.0916) among 4 (four) studies, marital status, risk difference 0.432 (95% CI = 0.177 to 0.687) within 6 (six) studies (P_het_ = 0.0001) and employment, risk difference, 0.39 (95% CI = 0.0136 to 0.775) among 5 (five) studies were independent risk factors significantly associated with HBV–HIV co-infection in pregnant mothers on ANC (*P* < 0.05) (Table 3, Figs. 12, 13, 14).

**Table 3:** Risk factor meta-analysis for HBV–HIV co-infection pooled risk difference estimation among ANC attendees

Risk factor	No	Analysis				
		Risk difference (95% CI)	*P* value	Z score	I^2^ (%) (95%CI)	P _het_
Age	4	− 0.517 (− 0.942 to − 0.0916)	0.017	2.382	91.44 (81.24 to 96.10)	< 0.0001
Marital status	6	0.432 (0.177 to 0.687)	0.01	3.321	81.57 (60.62 to 91.37)	= 0.0001
Educ. level	6	− .289 (− 0.764 to 0.185)	0.232	1.192	95.65 (92.80 to 97.38)	< 0.0001
Employment	5	0.39 (0.0136 to 0.775)	0.042	2.030	92.90 (86.39 to 96.26)	< 0.0001
Gravidity	2	0.386 (− 1.007 to 1.778)	0.587	0.543	95.1 (85.33 to 98.36)	< 0.0001

**Fig. 15:**
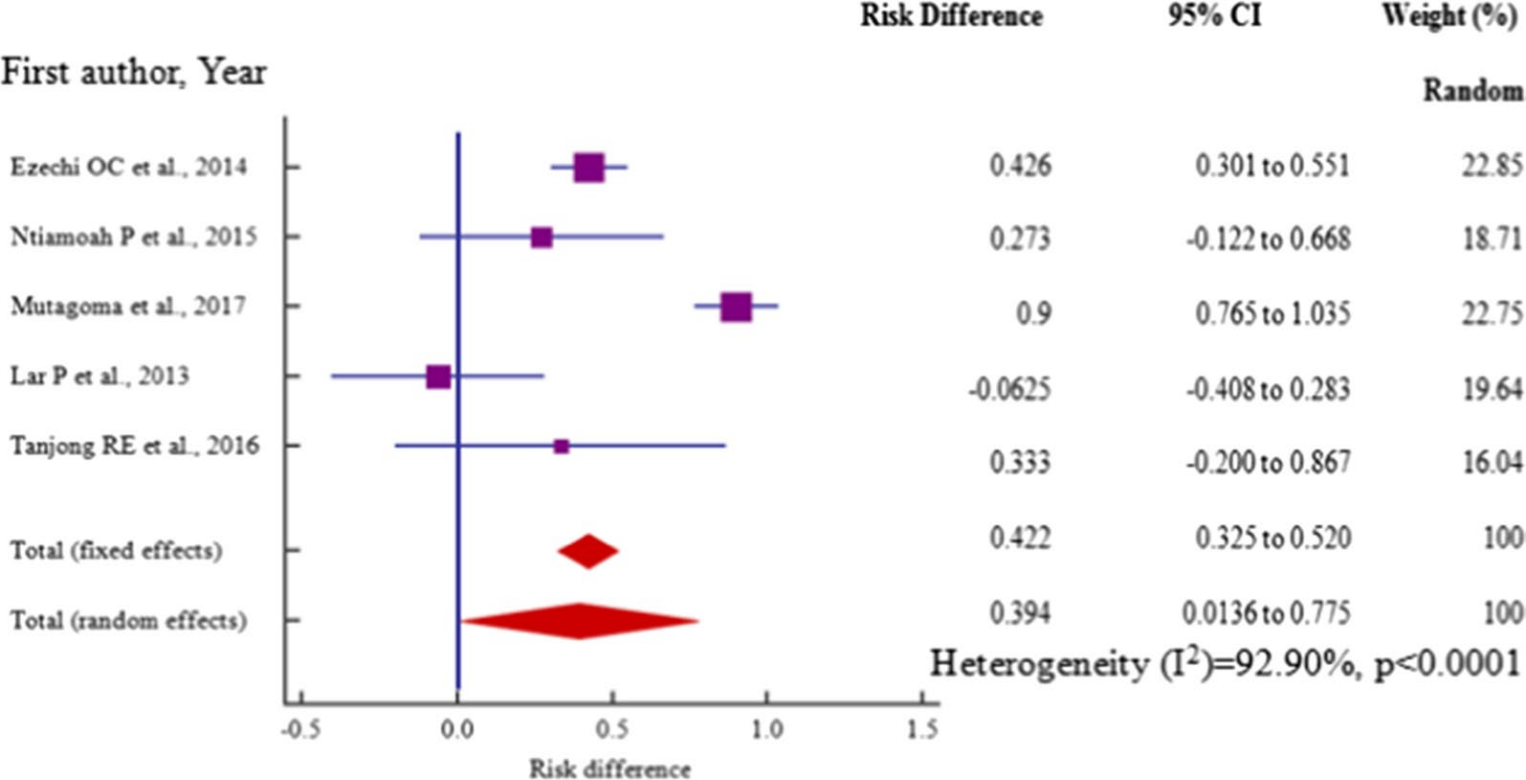
Forest plot of risk difference for HBV–HIV co-infection among employed and unemployed pregnant mothers from cross-section/cohort studies in sub-Saharan Africa from 2014 to 2019

**Fig. 16:**
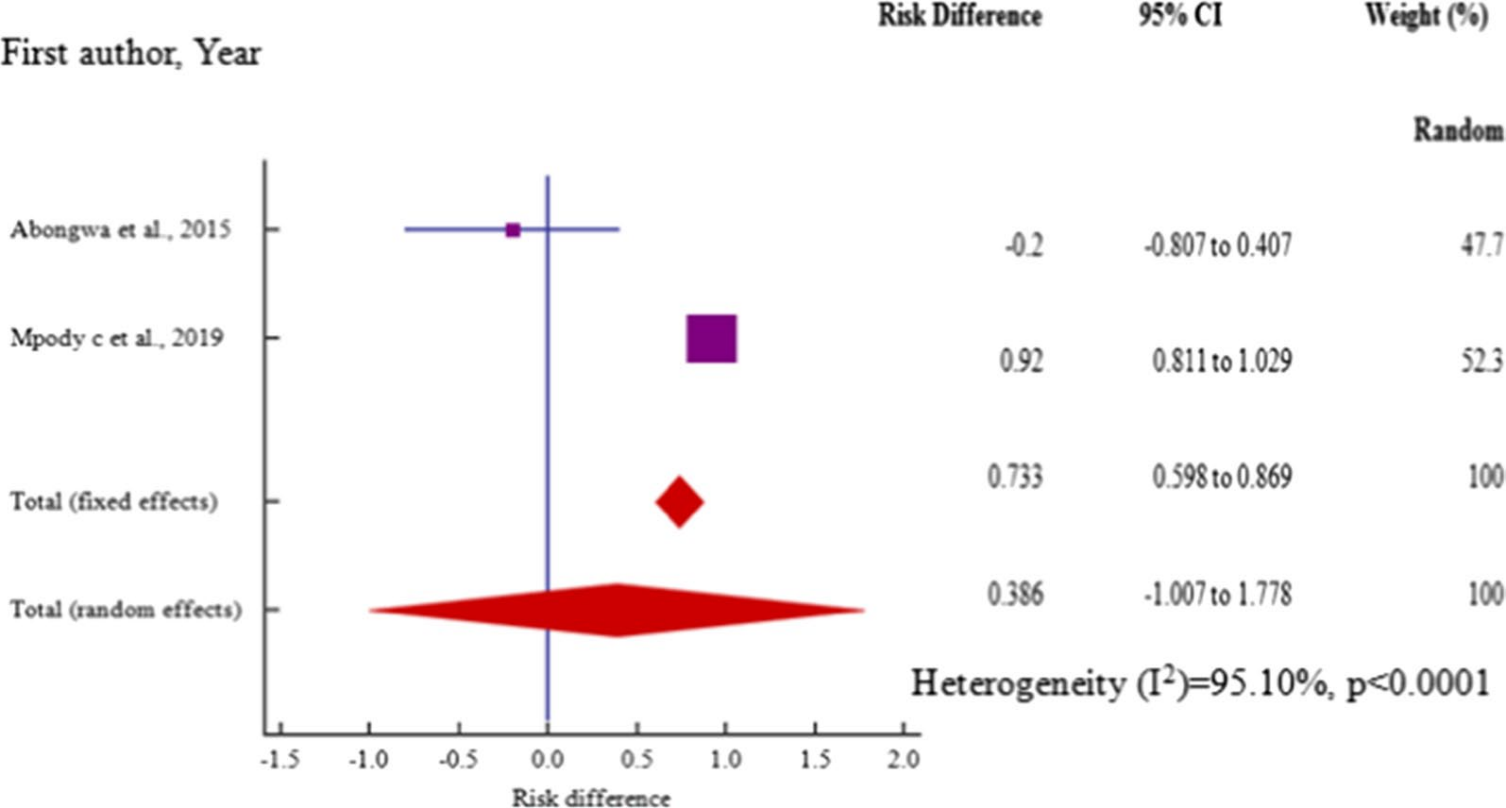
Forest plot of risk difference for HBV–HIV co-infection among the primagradida and multigravida mothers from cross-section/cohort studies in sub-Saharan Africa from 2014 to 2019

However, education level, risk difference −0.289(95% CI = −0.764 to 0.185) among 6 (six) studies and level of gravidity, risk difference 0.386(95%CI = −1.007 to 1.778) among 2 (two) studies were not significantly associated with HBV–HIV co-infection in pregnant mothers on ANC (*P* > 0.05) (Table 3, Figs. 15, 16). Moreover, all the studies included in our meta-analysis for the synthesis of data on the risk factors associated with HBV–HIV co-infection among the pregnant women on ANC had high heterogeneities (I^2^ > 81%, P_het_ ≥ 0.0001).

When we compared age, marital status, level of education, employment and magnitude of gravidity, with the prevalence of HBV–HIV co-infection among the pregnant mothers on ANC, we again obtained interesting associations. The HBV–HIV co-infection was significantly higher in pregnant mothers aged 25 years and above 75.635% (95% CI = 52.956 to 92.58%), married pregnant mothers 68.94% (95% CI = 57.89 to 83.689%) and among the employed mothers 70.756% (95% CI = 47.657 to 89.26%) (*P* < 0.001). However, there was no significant association between magnitude of gravidity with the prevalence of HBV–HIV co-infection (*P* = 0.0546) (Table 4). These observations are consistent with the increased risk of HBV–HIV co-infection among pregnant mothers in relation to age, marital status and employment record but not magnitude of gravidity as demonstrated by our meta-analysis of the risk difference (Table 3).

**Table 4:** Meta- analysis of the risk factors associated with HBV–HIV co-infection among pregnant mothers on ANC

Risk factor	Category	Number	Analysis	*P* value
			Proportion % (95%CI)	
*Age*				
	< 25 years	4	24.347 (7.42 to 47.074)	
	≥ 25 years	4	75.635 (52.956 to 92.58)	< 0.001*
*Marital status*				
	Married	6	68.94 (57.89 to 83.689)	
	Unmarried	6	24.36 (14.044 to 36.456)	< 0.001*
*Level of education*				
	Below secondary	6	35.52 (15.73 to 58.345)	
	Secondary & above	6	63.458 (40.44 to 83.617)	< 0.001*
*Employment*				
	Employed	5	70.756 (47.657 to 89.26)	
	Unemployed	5	25.193 (9.212 to 45.792)	< 0.001*
Gravidity	Primagravida	2	25.504 (0.757 to 83.0)	
	Multigravida	2	74.496 (16.99 to 99.243)	0.0546

^*^P value < 0.05 is considered statistically significant

### Meta-regression

**Fig. 17:**
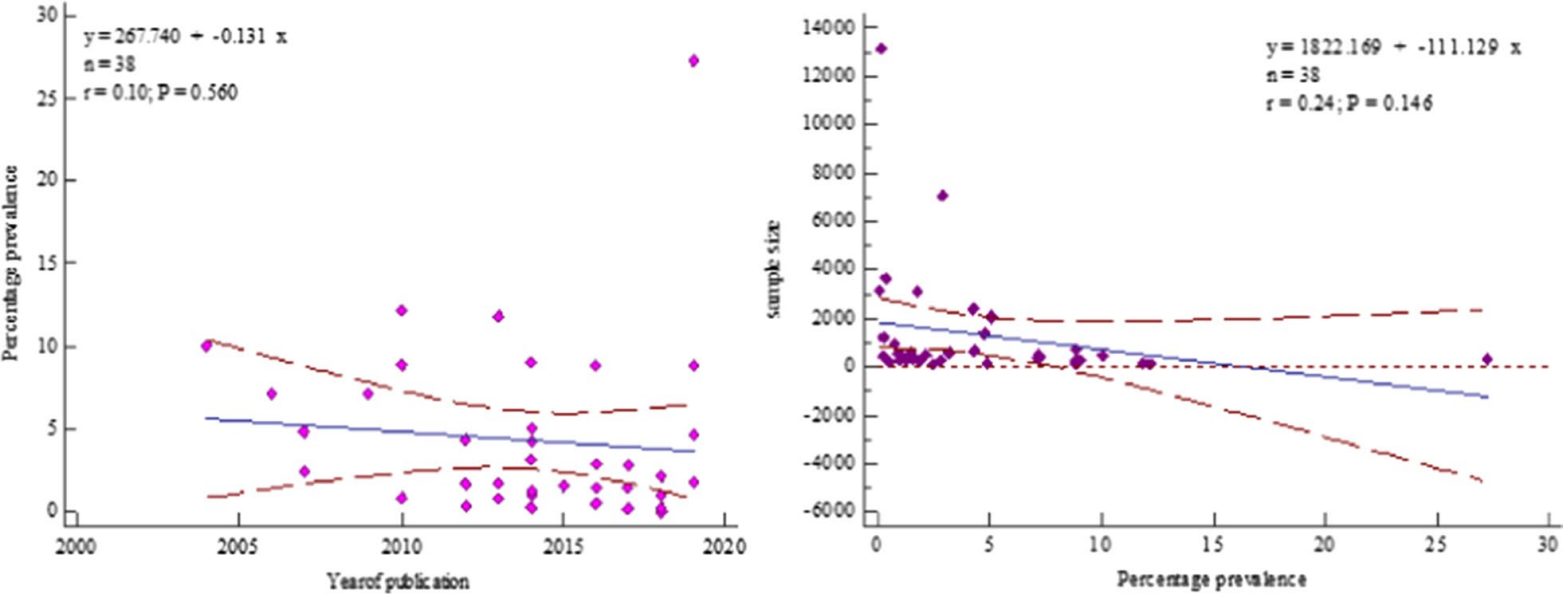
Meta-regression analysis by sample size and year of publication

Meta-regression analysis was performed to examine the continuous variables of sample size and prevalence HBV–HIV co-infection (*P* = 0.146) as well as year of publication (*P* = 0.560). The results were not significantly associated with HBV–HIV pooled prevalence (*P* > 0.05) (Fig. 17).

### Sensitivity analysis

**Fig. 18:**
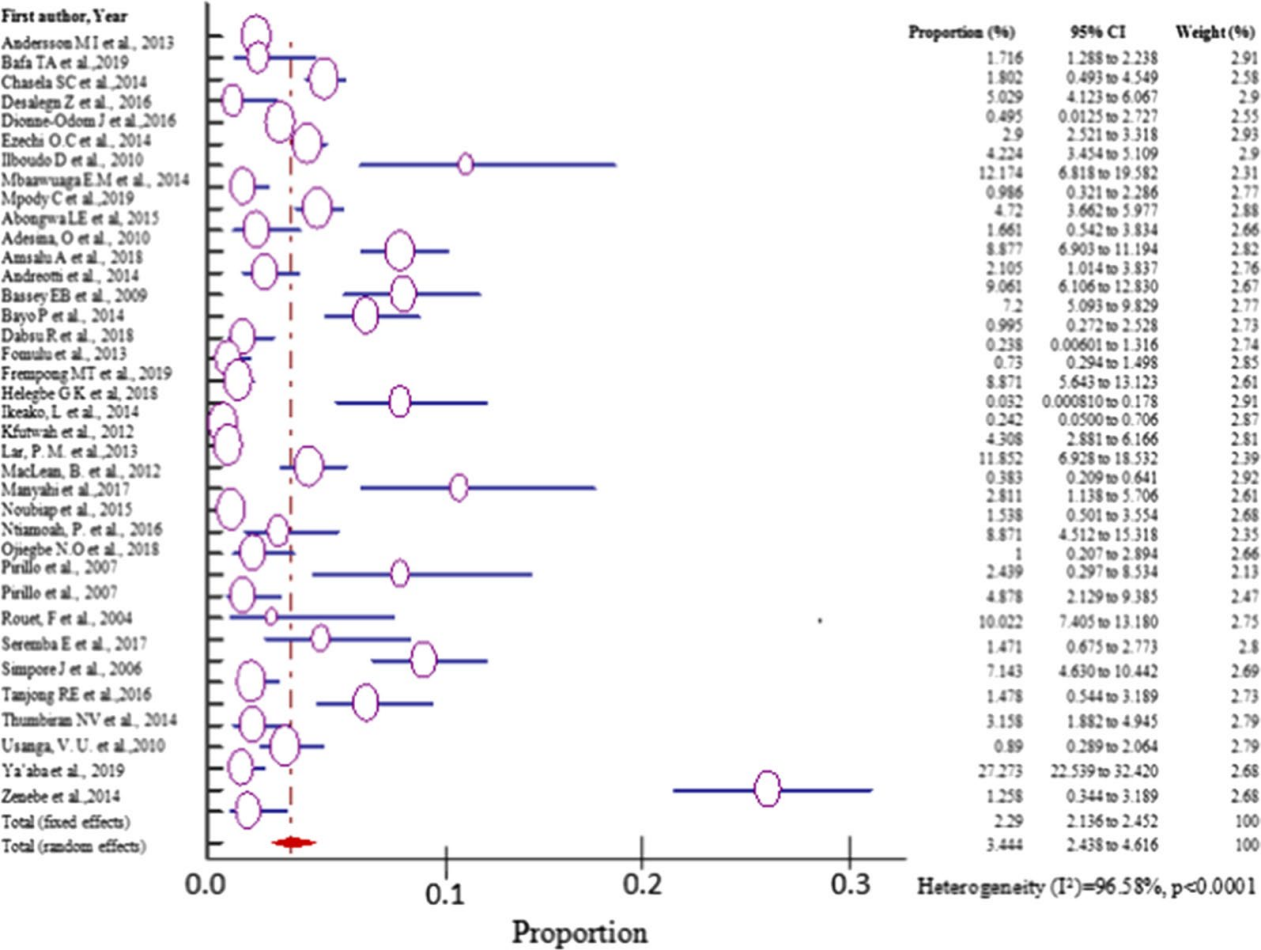
Sensitivity analysis after omission of the study with the largest sample size by the random effect model

We also performed a sensitivity analysis by removing one study with the largest sample size conducted in Rwanda [34]. For sensitivity analysis, the overall HBV–HIV pooled prevalence after omission, there was a slight increase to3.444% (95% CI = 2.438 to 4.616%) with heterogeneity I^2^ = 96.58% (95% CI = 95.92 to 97.13%), *P* < 0.0001(Fig. 18).

## Discussion

In our meta-analysis of 38 eligible studies, 36/38 (95%) were cross-sectional and 2/38 (5%) were from retrospective cohorts. Thus, the results of our study gave a reflection of HBV–HIV co-infection among pregnant women attending ANC at that point in time. The pooled prevalence estimate was 3.289% lower than the 4.9% HBV–HIV co-infection prevalence reported in Europe [62] among HIV positive pregnant women attending ANC and 4.6% reported in India among the 689 HIV infected pregnant women [63]. However, although the HBV–HIV co-infection prevalencies reported from Europe and India among the HIV positive cohort were lower than the prevalence of 8.321% in our meta-analysis in a similar cohort, the prevalence of HBV–HIV co-infection (8.7%) in Europe among HIV positive pregnant mothers with African decency [62] was in fair conformity with the result of our meta-analysis for the HIV positive cohort.

Results synthesized from studies that sampled mothers from the general population had generally lower HBV–HIV co-infection prevalence (2.152%) although relatively higher than the mean HBV–HIV co-infection prevalence reported in India (1.09%) from 36,379 pregnant women attending antenatal care from 15 antenatal care clinics [64], Iran (0.0%) among pregnant women [65], in Turkey (0.0004%) among 60,562 pregnant women [66], Turkey (0.1%) among 2548 pregnant women [67] and Cambodia (1.0%) [93]

The wide variation in prevalence of HBV–HIV co-infection among the pregnant mothers from SSA countries, Asia and Europe can be attributed to differences in implementation of control strategies, vaccination coverage [68], differences in endemicity of both HIV and HBV [1–4, 69], behavioral and cultural practices and differences in the sensitivity of the diagnostic methods employed [70]. Most importantly however, host genetic factors [71, 72, 85] and the infecting genotypes [15, 73–76] could be more influential factors in the risk of infection to HBV creating differences and similarities in the burden of HBV–HIV co-infection over a wide ethnic and geographical divide. Among the host genetic factors, polymorphisms in the cytokine promoter gene [94, 95] Vitamin D Receptor [96, 97] and Human Leucocyte Antigen [98, 99] influence the susceptibility to viral infections yet these are individual and population specific [94–99]. Similarly, available evidence has implicated genotype A to be the most prevalent in HBV–HIV co-infection [77]. Moreover, epidemiological studies on HBV disease profile have shown that infection with genotype A is pandemic [78] and predominant in most of the sub-Saharan African countries [79]. Consequently, the similarity in the observed burden of HBV–HIV co-infection among pregnant mothers from one region to another could be attributed to the universal geographical distribution of HBV genotype A.

The pooled prevalence of HBV–HIV co-infection estimate was highest in West Africa (5.155%) followed by Central Africa (2.441%), South Africa (2.317%) and least in East Africa (2.168%). The high burden of HBV–HIV co-infection among the pregnant women in West Africa can partly be explained by the target population used in many West African studies included in our meta-analysis. Out of the 12 studies that targeted HIV positive mothers, five (5/12) were conducted from West Africa (42%) and only 3/12 (25%) for each of Central Africa and East African countries. Moreover, 3/7 eligible studies (43%) included in our meta-analysis from Central Africa targeted HIV positive pregnant mothers but only 3/13 (23.1%) from East African countries. The shared route of transmission for both viruses [80] and the compromised immunity due to HIV infection partly explains the high burden of HBV–HIV co-infection among the HIV positive mothers. Most importantly, the global highest burden of HBV–HIV co-infection has been reported from Western African countries [81, 82]. The pooled prevalence estimate of HBV–HIV co-infection among the pregnant mothers in South Africa reported in our meta-analysis should be interpreted with caution since only two eligible studies were included in our meta-analysis [14, 54]. Consequently, we were limited in our ability to make a comprehensive regional comparison of HBV–HIV co-infections among the pregnant mothers on ANC. For example, in southern sub-Saharan Africa where estimated HIV prevalencies are higher than rest of the continent with majority of the countries having a prevalence ≥ 20% [100], only one country had data on the HBV–HIV co-infection among pregnant mothers [14, 54]. On the other hand, western sub-Saharan Africa with apparently low HIV prevalence in the general population posted the highest HBV–HIV co-infection prevalence among the pregnant women [18, 39–53]. This can be attributed to differential HIV burden in this region by sex with women having almost twice as high HIV prevalence than men [101]. Moreover, women continue to carry a higher HIV burden in SSA [102] accounting for 58% of the people living with HIV (PLWH) in our region [103].

The differences in regional burden of HBV–HIV co-infection among the pregnant mothers can in addition be explained by differences in endemicity of the viruses [69], circulating genotypes [83], risk factors to infection [84], host genetic factors [85], vaccination coverage [68] as well as implementation of control and prevention strategies [86]. However, the HBsAg detection method did not confound the results of our meta-analysis (*P* > 0.05).

Regarding the year of publication, recently published studies reported significantly lower pooled prevalence estimate of 2.252% compared to earlier studies with pooled prevalence of 6.386% (*P* < 0.001). The decrease in HBsAg positivity rate among pregnant women on ANC observed in recent publications in our meta-analysis has also been reported in Turkey from a period of 2013 to 2016 [87] and from 1995 to 2015 [88]. This could be probably attributed to improved awareness of both viruses [68] as well as introduction of national immunization programs.

Age, marital status, level of education and employment were significantly associated with HIV–HBV co-infection among pregnant women in our meta-analysis (*P* < 0.001). Therefore, interventions to manage co-infections with HBV and HIV among pregnant mothers should target those who are married, employed, educated and aged between 25–30 years. This is in conformity with the findings by Mohammad et al*.* [89]*,* who reported that, the marital status, occupation and age were significant risk factors for HIV–HBV co-infections among HIV–infected patients in Iran. Similarly, a study on HBV–HIV co-infected pregnant women in Europe reported a significant association of age with HBV–HIV co-infection with women aged 25–29 years being at higher risk of HBV–HIV co-infection [62] in conformity with our meta-analysis. The high sexual activity in the age group ≥ 25 years [90] has been implicated in the high prevalence of HBV–HIV co-infection since both HBV and HIV are sexually transmitted infections [91]. Perhaps, there is also an unmet need pertaining being faithful to each partner in marriages and among those in sexual relationships increasing the risk of transmitting both HIV and HBV in sub-Saharan African in general and pregnant women in particular. Most importantly however, despite the recommendation for administration of HBV vaccine to all infants in 1991 by the Advisory Committee on Immunization (ACIP) in the United States [104], the implementation of the HBV vaccination program as part of the extended program on immunization (EPI) in Africa started in 1995 [105]. Thus pregnant mothers born before 1995 were not immunized against HBV in most of the SSA countries increasing the burden of HBV–HIV co-infection in those above 25 years as opposed to those below 25 years who were born after 1995.

Whereas gravidity was not statistically associated with significant risk of getting HIV–HBV co-infection (*P* = 0.0546) in our meta-analysis, having two or more pregnancies was identified as risk factor associated with HBV and HIV co-infection in the study on HBV infections among pregnant women in Arab countries [92].

## Conclusion

This meta-analysis has provided up-to-date information on the HBV–HIV co-infection status among pregnant mothers on antenatal care in sub-Saharan Africa. Moreover, most of the studies are recently published (2011to 2019). Our meta-analysis has identified differences in burden of the co-infection with both viruses from different sub-regions. We have also highlighted the risk factors to HBV–HIV co-infection among pregnant mothers in sub-Saharan. The overall pooled prevalence was higher than prevalencies reported in Europe and Asia suggesting an unmet need for effective control strategies increasing the risk of having a pool of new born with HBV and perhaps HIV. However, HBV–HIV co-infection among pregnant mothers in HIV positive cohort subgroup pooled prevalence estimate in our meta-analysis is comparable to those reported in previous studies due to the shared route of transmission for both viruses and the discrepancies are due to differences in endemicity, control strategies, infecting genotypes and host immunity. We recommend that HBV screening be prioritized during antenatal visits as is the case with HIV in sub-Saharan Africa because of the observed burden among the HIV positive mothers in our meta-analysis.

### Limitations

Published literature in peer reviewed journals on HBV–HIV co-infection among pregnant mothers attending antenatal care is scanty. The 38 eligible studies included in our meta-analysis were conducted from only 13 SSA countries. Therefore, the HBV–HIV co-infection pooled prevalence estimate among pregnant mothers of 3.3% may not be representative of the burden of the co-infection in SSA countries. Moreover, HBV screening in many SSA countries is not routine and therefore its epidemiology is not well documented.

## Data Availability

All data generated or analyzed during this study are included in this published article.

## Abbreviations

SSA: Sub-Saharan Africa
HIV: Human immunodeficiency virus
HBV: Hepatitis B virus
ANC: Antenatal care
PRISMA: Prefered reporting items for systematic reviews and meta-analyses
HBeAg: Hepatitis B pre-core Antigen
HBcAg: Hepatitis B core antigen
HBsAg: Hepatitis B surface antigen
cART: Combined antiretroviral therapy
MTCT: Mother-to-child-transmission
CD: Cluster of differentiation
AAS: Abbott ARCHITECT system
VCIA: Vitros chemiluminescence immunoassay
EIA: Enzyme immuno assay
ELISA: Enzyme linked immuno assay
RDT: Rapid diagnostic test
QS: Quality score
